# Retrospective Seroprevalence of Orthopoxvirus Antibodies among Key Populations, Kenya

**DOI:** 10.3201/eid3009.240510

**Published:** 2024-09

**Authors:** Kristi Loeb, Kieran A. Milner, Candice Lemaille, Brielle Martens, Derek Stein, Julie Lajoie, Souradet Y. Shaw, Anne W. Rimoin, Placide Mbala-Kingebeni, Nicole A. Hoff, Ryan S. Noyce, Keith R. Fowke, Joshua Kimani, Lyle McKinnon, Jason Kindrachuk

**Affiliations:** University of Manitoba, Winnipeg, Manitoba, Canada (K. Loeb, K.A. Milner, C. Lemaille, B. Martens, D. Stein, J. Lajoie, S.Y. Shaw, K.R. Fowke, J. Kimani, L. McKinnon, J. Kindrachuk);; Cadham Provincial Laboratory, Winnipeg (D. Stein);; University of Nairobi, Nairobi, Kenya (J. Lajoie, K.R. Fowke, J. Kimani, L. McKinnon);; University of California–Los Angeles, Los Angeles, California, USA (N.A. Hoff, A.W. Rimoin);; Institut National de Recherche Biomédicale, Kinshasa, Democratic Republic of the Congo (P. Mbala-Kingebeni);; University of Alberta, Edmonton, Alberta, Canada (R.S. Noyce)

**Keywords:** orthopoxvirus, viruses, sexually transmitted infections, zoonoses, seroprevalence, antibodies, Kenya

## Abstract

We identified a cluster of mpox exposures among key populations in Kenya through retrospective serologic screening. We identified strong seropositivity among sex workers and gay, bisexual, and other men who have sex with men. These findings demonstrate the need for increased mpox surveillance among mpox-endemic and mpox-endemic–adjacent regions in Africa.

Mpox (formerly monkeypox) is a zoonotic viral disease caused by monkeypox virus (MPXV) that saw rapid geographic expansion resulting in a global epidemic in 2022 (*1*). MPXV consists of 2 distinct clades (clades I and clade II); clade I infections are associated with greater disease severity (*1*,*2*). Clade II MPXV is subdivided into 2 subclades (clades IIa and IIb); clade IIb is linked to the global epidemic (*3*,*4*). Although zoonosis has been the primary driver of human infections and outbreaks have occurred primarily in tropical forest regions within mpox-endemic countries, during the 2022 epidemic, >90% of infections were linked to secondary transmission, mainly through close, intimate (often sexual) contact. Historically, mpox has primarily affected younger populations; however, most of those mpox cases were associated with clade I (*3*). The average age of clade IIb mpox case-patients during the 2022 epidemic was >30 years; most were male (98.7%) and identified as gay, bisexual, and other men who have sex with men (GBMSM) (84%) (*4*). During the 2022 epidemic, common clinical characteristics for mpox included fever, physical asthenia or lethargy, lymphadenopathy, and rash; atypical lesion location also was noted.

MPXV reemerged in Nigeria in 2017 and resulted in ongoing endemic circulation. In contrast to historic mpox, disease has been more prevalent in urban regions and among adults (*5*). Human-to-human transmission and a high proportion of genital ulcers also were noted. More recently, transmission of clade I MPXV associated with intimate contact has been reported in the Democratic Republic of the Congo (DRC), including geographic expansion and cases in multiple large urban areas (*6*).

MPXV genome sequencing demonstrated linkages between reemergence in Nigeria and global expansion in 2022 (*7*,*8*). Given the reemergence of mpox in Nigeria with sustained nonzoonotic transmission, ongoing global circulation, rapid expansion in DRC, and acquisition associated with intimate contact, an urgent need exists for expanded mpox surveillance in Africa, particularly among key populations at increased risk for infection. Given the paucity of mpox surveillance data and the role of Nairobi (capital city of Kenya) as a major economic and transit center in Africa, we undertook retrospective orthopoxvirus (OPXV) serologic screening that focused on key populations.

## The Study

We used historic serum samples from male and female sex workers (n = 656) enrolled at the Sex Workers Outreach Program (SWOP) in Nairobi, Kenya (*9*). We assayed samples for IgG seropositivity by using a modified ELISA assay with UV-inactivated vaccinia virus (VACV). We screened samples by using a 1:50 dilution (*10*). We collected samples during 2013–2018; age range of participants was 19–69 years at sample acquisition (Table). Female sex workers accounted for 72.3% (474/656) of our sample population, followed by other (non–sex workers or nonidentified; 18.4% [122/656]) and GBMSM (9.3% [61/656]). We defined seropositivity on the basis of absorbance values >3 SDs above baseline. Most (76.7% [503/656]) samples were provided by persons 20–55 years of age; we found the highest percentage of seropositive samples among participants 20–39 years of age (37.4% [37/99]) and 40–55 years of age (36.4% [36/99]) among all seropositive samples tested. We detected 89% seropositivity among persons living with HIV; however, HIV positivity was high (85.8% [563/676]) among the sample population (Figure; Appendix Figure).

**Table:** Demographic data for all participants in a retrospective study of seroprevalence of orthopoxvirus antibodies among key populations, Nairobi, Kenya, 2013–2018*

Characteristic	Human orthopoxvirus seropositivity		Total (%)
	Positive	Negative	
Age group, y			
<19	0	1 (0.2)	1 (0.2)
20–39	37 (37.4)	188 (33.8)	225 (34.3)
40–55	36 (36.4)	242 (43.4)	278 (42.4)
56–65	8 (8.1)	60 (10.8)	68 (10.4)
>65	2 (2.0)	5 (0.9)	7 (1.1)
Unknown	16 (16.2)	61 (11.0)	77 (11.7)
Total	99	557	656
Sample collection year			
2013	1 (1.0)	7 (1.3)	8 (1.2)
2014	5 (5.1)	55 (9.9)	60 (9.1)
2015	3 (3.0)	15 (2.7)	18 (2.7)
2016	3 (3.0)	13 (2.3)	16 (2.4)
2017	61 (61.6)	277 (49.7)	338 (51.5)
2018	26 (26.3)	190 (34.1)	216 (32.9)
Total	99	557	656
HIV status			
Negative	11 (11.1)	82 (14.7)	93 (14.2)
Positive	88 (88.9)	475 (85.3)	563 (85.8)
Total	99	557	656
Key population			
Female sex worker	63 (63.6)	411 (73.8)	474 (72.3)
GBMSM	15 (15.2)	46 (8.3)	61 (9.3)
Non–sex worker	21 (21.2)	100 (17.9)	121 (18.4)
Total	99	557	656

*Participants were enrolled at the Sex Workers Outreach Program (SWOP) in Nairobi, Kenya. Values are no. (%). Seropositivity determined by ELISA using UV-inactivated vaccinia virus. GBMSM, gay, bisexual, and other men who have sex with men.

**Figure:**
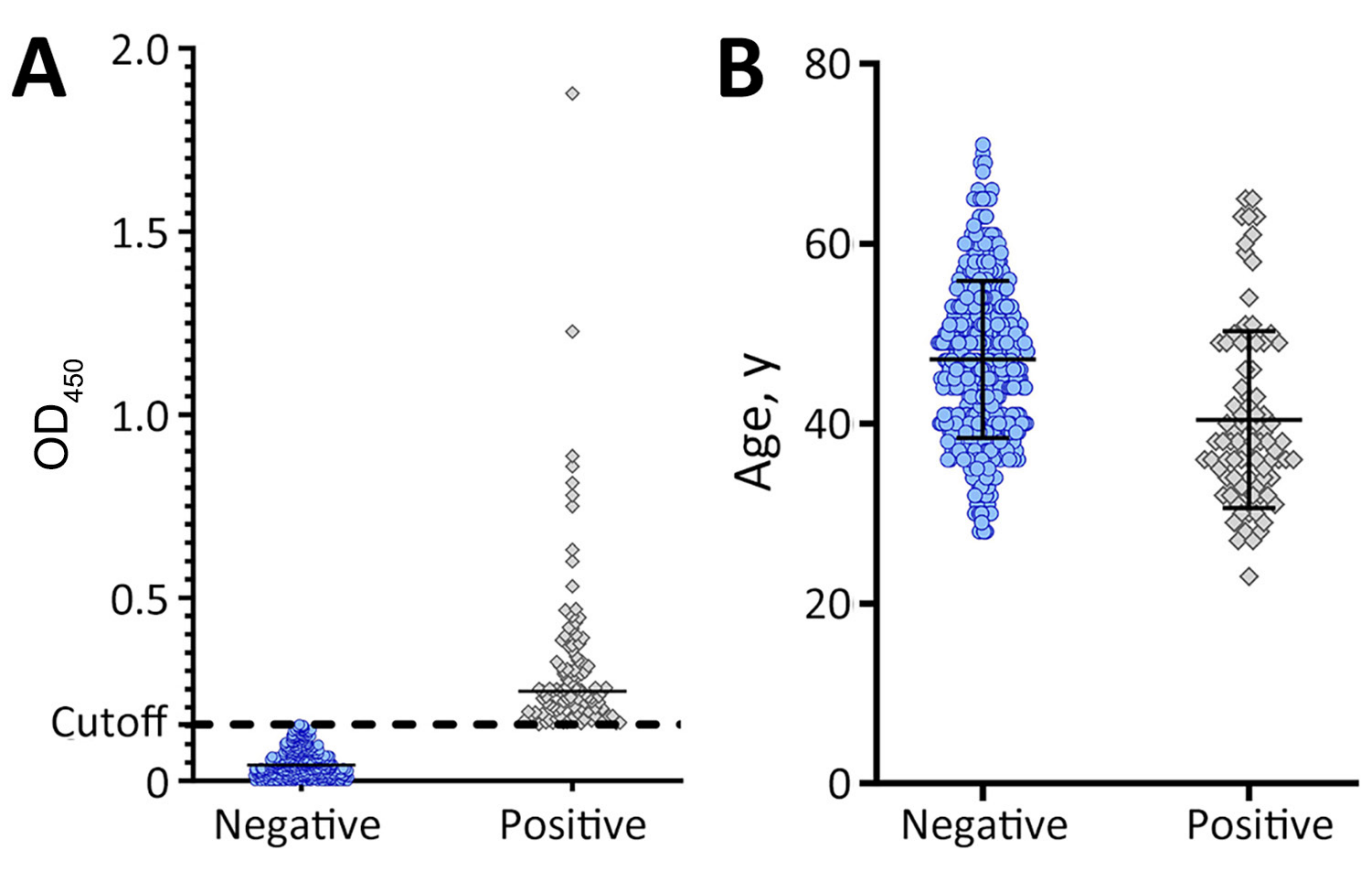
Retrospective assessment of orthopoxvirus antibody seropositivity among key populations, Nairobi, Kenya, 2013–2018. Orthopoxvirus seropositivity was assessed in banked samples acquired from 792 sex workers. A) Seropositivity was determined by ELISA using UV-inactivated vaccinia virus. Cutoff line indicates baseline seropositivity. Positives were determined as 3 SDs above the cutoff. Horizontal bar indicates median. B) Age distribution across all seropositive and seronegative samples. Horizontal bars indicate median with upper quartile above, lower quartile below. OD_450_, optical density at 450 nm.

We next selected 111 samples from the ELISA screen for subsequent analysis by the OPXV IgG Panel (Meso Scale Discovery, https://www.mesoscale.com), which includes 5 MPXV antigens: A29, A35, B6, E8, and M1 and their corresponding VACV orthologs. We tested samples by using 1:500 dilutions. Cross-reactivities for the assay range from 198% (MPXV A35R and VACV A33R) to 43.4% (MPXV E8L and VACV D9L), and lower limits of quantitation ranged from 0.021 to 0.058 AU/mL. We selected 86 samples that met seropositive criteria from the ELISAs (>3 SDs above baseline) and 25 seronegative or borderline samples. We blinded samples during testing and repeated sampling on a subset of blinded samples for validation. Average age for participants from whom samples were taken was 49 years (range 29–74 years); samples were from female sex workers, GBMSM, and non–sex workers. We detected OPXV seropositivity across all age groups in our sample cohort, and had strong signals (>10,000 AU/mL) within all age groups, including persons born after cessation of the global smallpox vaccination program (*11*). OPXV-positive samples included 5 samples from persons in the 20–39-year age group, with calculated concentrations of >1,000 AU/mL. Our data suggest MPXV exposure among groups already at increased risk for infection in Kenya.

## Conclusions

The reemergence of clade II MPXV in Nigeria in 2017, followed in 2022 by the rapid global expansion of clade II MPXV across non–mpox-endemic regions and the continued expansion of the current clade I outbreak in DRC, highlight the need for ongoing mpox surveillance. Given the geographic proximity and expansive shared borders of multiple countries in East Africa with DRC, frequent movement of persons across those regions, and the role of Nairobi as a commercial and tourism center, expanded mpox surveillance is needed urgently.

Although MPXV transmission between humans has historically occurred through close contact with infected persons (*2**,**10**–**12*), transmission during the global mpox epidemic was strongly linked to close, intimate (including sexual) contact. Transmission through close and sexual contacts have been observed during the ongoing mpox outbreak in DRC.

Given the risk for further expansion of MPXV in Africa, we screened for indications of historic mpox exposures in key populations at increased risk for infection in Kenya. Our data suggest that unreported MPXV exposures have occurred within key populations. The smallpox vaccination campaign in Kenya ended in 1972 (although vaccinations may have occurred later), and only importation-related cases were reported after 1970 (*11*). Thus, seropositivity against VACV and MPXV in persons <52 years of age in our sample population is strongly indicative of environmental exposure to an orthopoxvirus independent of variola virus. Although camelpox virus has been reported among camels in northern Kenya, few human orthopoxvirus infections have been reported in the region and zoonosis is rare (*12*). Camelpox cannot be discounted given antigenic similarities to other human orthopoxviruses; however, the clustering of seropositivity among key populations in our study suggests an alternative virus source. No other orthopoxviruses are known to infect humans in Kenya; thus, our serologic data suggest potential MPXV exposure. 

Our findings highlight the need for expanded and sustained mpox surveillance that includes non–mpox-endemic regions close to areas with active mpox outbreaks. In addition, stigmatization and fear of repercussions or persecution encountered by sex workers and GBMSM communities may have also limited the historical identification of mpox in non–mpox-endemic regions of sub-Saharan Africa. 

One limitation of our study stems from the antigenic similarity among human orthopoxviruses. The MPXV antigens we used in this study have VACV orthologs, and the seropositivity we detected cannot definitively identify prior mpox nor differentiate MPXV clades. However, given the seropositivity among persons within key populations linked to sex work and dense sexual networks, including persons born after the global smallpox vaccination program ended, our data support expanded mpox surveillance in regions proximal to mpox-endemic areas. 

In summary, our data suggest that mpox introduction among sex workers in Kenya probably occurred before identification of MPXV reemergence in Nigeria, the 2022 epidemic, and the ongoing outbreak in DRC. The source of these exposures could have included undiagnosed mpox circulation and introduction from either Central or West Africa, considering the lack of clade-specific determination through serologic screening. Thus, a definitive need exists to establish enhanced surveillance for groups at elevated risk for MPXV infection in Kenya and surrounding regions.

Appendix Additional information about retrospective seroprevalence of orthopoxvirus antibodies among key populations, Kenya.
