# Supplementary material for: Retrospective Seroprevalence of Orthopoxvirus Antibodies among Key Populations, Kenya
Source: Emerg Infect Dis. 2024 Sep;30(9):1944–7. doi: 10.3201/eid3009.240510 (PMC11347004; doi:10.3201/eid3009.240510)
Supplement: Appendix — Additional information about retrospective seroprevalence of orthopoxvirus antibodies among key populations, Kenya. [file 24-0510-Techapp-s1.pdf]

Article DOI: https://doi.org/10.3201/eid3009.240510

Retrospective Seroprevalence of Orthopoxvirus Antibodies among Key Populations, Kenya

Appendix

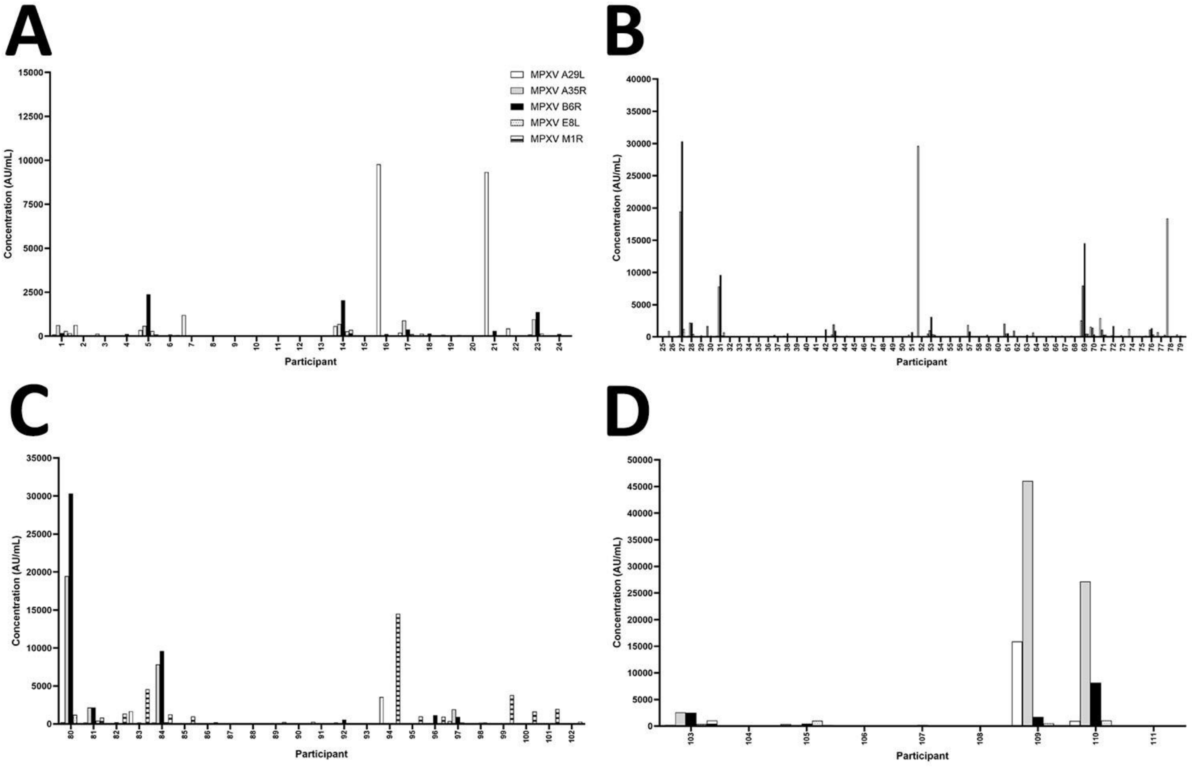

Appendix Figure. Multiplex serology screening of orthopoxvirus seropositive ELISA samples against MPXV and VACV antigens. Orthopoxvirus seropositive samples identified using ELISA screening with UV-inactivated VACV were subsequently screened against multiplexed MPXV and VACV antigens. Calculated concentrations (AU/mL) are presented across the following age brackets: A) 20–39 years; B) 40–55 years; C) 56–65 years; D) >65 years.
